# Psychological Violence and Attitudes Towards Love in Intimate Partner Relationships Among University Students

**DOI:** 10.3390/bs16050763

**Published:** 2026-05-13

**Authors:** Laura Pérez-Díaz, Macarena Blázquez-Alonso, María Elena García-Baamonde Sánchez, Carlos Barbosa-Torres, Juan Manuel Moreno-Manso

**Affiliations:** Departament of Psychology, Faculty of Education and Psychology, University of Extremadura, 06006 Badajoz, Spain; laurapd@unex.es (L.P.-D.); mgarsan@unex.es (M.E.G.-B.S.); carlosbarbosa@unex.es (C.B.-T.); jmmanso@unex.es (J.M.M.-M.)

**Keywords:** psychological violence, attitudes towards love, intimate partner relationships, university students

## Abstract

Psychological violence is the most frequent form of aggression in intimate partner relationships. It is characterised by its subtle nature, which hinders detection and intervention. Understanding this phenomenon requires examining attitudes toward love, as they influence how violent behaviours are perceived and legitimised. The aim of this study was to identify manifestations of psychological violence and love attitudes among university students, to analyse the relationship between these variables, and to determine the contribution of love attitudes in explaining the variability in psychological violence. An observational cross-sectional study included 1118 university students aged 17–23 years (*M* = 20.17; *SD* = 1.66). The Questionnaire on Psychological Violence in Intimate Partner Relationships (CVPPar) and the Short Love Attitudes Scale (LAS) were administered. The findings revealed variability in both psychological violence and love attitudes, as well as significant associations between the two. In particular, the Eros, Ludus, and Mania styles showed the strongest associations with most of the manifestations examined. Regression analyses indicated that love attitudes significantly contributed to explaining variance in psychological violence, with explained variance ranging from 34% to 43%, indicating moderate explanatory power. This study concludes that attitudes towards love are associated with psychological violence, emphasising the importance of early psychoeducational interventions.

## 1. Introduction

Psychological violence within intimate relationships represents a global public health concern. According to the World Health Organisation, approximately 27% of women who have been in a relationship have experienced some form of violence by an intimate partner during their lifetime (WHO, 2021). Furthermore, international research consistently identifies psychological violence as one of the most prevalent forms of this phenomenon. This persistence is associated with deeply rooted cultural and social structures, including gender norms that legitimise the subordination of women and expectations regarding family roles, as well as longstanding dynamics of inequality, such as systemic gender discrimination and the concentration of power within patriarchal structures (Huang et al., 2024).

Psychological violence is defined as any action or behaviour intended to inflict emotional, cognitive, or behavioural harm, thereby affecting an individual’s physical and mental integrity and well-being. Its progressive nature and the difficulty of its identification hinder victims’ ability to recognise the situation, which in turn complicates both clinical and social detection and intervention (Páramo et al., 2021; Tutiven-Abad et al., 2022). These behaviours may take both overt forms, such as threats, verbal aggression, or humiliation, and more covert forms that are harder to detect, including emotional manipulation, indifference towards the victim’s needs, minimisation of suffering, or the attribution of responsibility for conflicts to the victim (Oliveira & Loayza, 2022; Vidal et al., 2024; Villavicencio-Aguilar & Jaramillo-Paladinez, 2020; Yastıbaş-Kaçar et al., 2024; Zhi et al., 2021). In this regard, Taverniers (2001) expands this conceptualisation through a more detailed typology distinguishing (a) explicit interpersonal behaviours such as devaluation, hostility, and intimidation; (b) relational attitudes such as indifference or apparent benevolence; (c) strategies of attributing responsibility to the victim through accusations or insinuations; and (d) normative imposition behaviours that restrict autonomy within the relationship.

Furthermore, the complexity of psychological violence in intimate partner relationships is reflected in the diversity of its manifestations and the difficulty of its detection. Moreover, social normalisation contributes to its invisibility through cultural attitudes, institutional practices, and interpersonal dynamics that minimise or tolerate such behaviours (Fedele et al., 2019; Martín & Moral, 2019).

From an integrative theoretical perspective, this phenomenon can be explained through socio-cognitive models of relationships and the ecological theory of human development, which emphasise the continuous interaction between individual beliefs and attitudes, relational patterns, sociocultural norms, and processes associated with developmental stage. Taken together, these frameworks allow psychological violence to be understood not as an isolated phenomenon, but as the result of dynamic processes in which cognitions about love and relationships are shaped, learned, and activated within specific social contexts.

Understanding these dynamics requires consideration of the cultural frameworks that shape intimate partner relationships. The persistence of cultural norms, institutional structures, and patriarchal practices fosters the continuity of violence. Discourses that exalt the romantic ideal of absolute devotion or self-sacrifice normalise abusive behaviours by justifying the victim’s subordination, minimising emotional harm, and obscuring dynamics of control. These expectations are reinforced through cultural representations in the media and literature, as well as through social norms regarding gender roles, thereby constructing a romantic ideal that hinders the recognition of violence (Guerra-Marmolejo et al., 2021; Sánchez-Hernández et al., 2020).

Despite advances in gender equality, an asymmetrical model of love persists, characterised by emotional dependence, exclusivity, and absolute devotion (Mingo, 2020; Ortega et al., 2024). Gender stereotypes legitimise controlling dynamics within intimate partner relationships, while the internalisation of unconditional love hinders victims’ ability to question abusive behaviours (Bringas-Molleda et al., 2017; Douadi et al., 2024).

Love styles constitute a key theoretical framework for understanding these dynamics. Lee (1973) described the Eros style, centred on passion and emotional fusion, as the archetype of traditional romantic love (Flores et al., 2019; Lisham, 2017). Unrealistic expectations derived from this style generate frustration and distress when they are not fulfilled (Aunola et al., 2021). Recent research indicates that such discrepancies may manifest in subtle forms of psychological violence that become normalised within everyday interactions (Ablana et al., 2024; Ramírez-Carrasco et al., 2024).

The Ludus style, oriented toward pleasure and emotional detachment, can lead to the instrumentalization of the partner and the prioritisation of individual gratification over shared well-being (Rafiezadeh & Zarehneyestanak, 2021; Yıldız & Eldeleklioğlu, 2021). Its combination with Eros gives rise to the Mania style, characterised by possessiveness, jealousy, and control, and associated with low self-esteem, emotional instability, and dependent or anxious attachment (Agus et al., 2021; Alcalá et al., 2021; Blanchard & Fino, 2023; Faraji & Başçelik, 2023; C. Hendrick & Hendrick, 1986; S. S. Hendrick & Hendrick, 2006; Jonason et al., 2020).

Other love styles, such as Storge, Agape, and Pragma, should be understood respectively as forms of love based on friendship and emotional stability (Storge), altruism and selfless devotion (Agape), and rationality and practical compatibility in partner selection (Pragma), although in certain relational contexts they may be indirectly associated with covert control dynamics. Storge may lead to dominant behaviours driven by fear of loss (Díaz et al., 2019; Neto & Da Concelçao, 2024; Vedes et al., 2016). Agape may justify abuse in the name of sacrifice or care for the partner (Galicia et al., 2013; Robles et al., 2021). Pragma may reinforce relational inequality when one partner maintains dominance to preserve the stability of the relationship (Heron et al., 2022; Pilkington et al., 2021; Ruiz-Eugenio et al., 2020). Examining these styles allows for an understanding of how certain conceptions of love contribute to the emergence and perpetuation of psychological violence.

From this perspective, love styles can be conceptualised as socio-cognitive schemas that guide the interpretation of a partner’s behaviours and responses to them, while the sociocultural and developmental context modulates their expression.

In this sense, love styles provide a useful framework for analysing the emergence and persistence of psychological violence, particularly during emerging adulthood, as is the case in university populations. This developmental stage represents a period of heightened vulnerability due to ongoing identity formation, emotional consolidation, and the development of social skills (Fortin et al., 2012; Lascorz et al., 2020; O’Leary, 1999; Perles et al., 2019, 2022). During these developmental phases, behaviours such as control, jealousy, or threats may be misinterpreted as expressions of love, due to cultural beliefs that normalise possessiveness and emotional dependence within romantic relationships (Muñoz-Galiano et al., 2024; Muñoz-Ponce et al., 2020; Perles et al., 2022; Safranoff, 2017).

In the university context, these dynamics acquire particular relevance, as this stage involves the consolidation of early affective relationships during emerging adulthood, characterised by a combination of increasing autonomy, relational exploration, and the persistence of idealised romantic beliefs. This may favour the emergence of subtle forms of psychological violence that tend to be normalised or not recognised as such.

Within this framework, the joint analysis of attitudes toward love and the different manifestations of psychological violence emerges as a key area of interest, as it enables a better understanding of how specific cognitive–affective schemas are associated with dysfunctional relational dynamics. The examination of this relationship therefore has important implications for the design of preventive strategies and psychoeducational programmes aimed at promoting healthy romantic relationships, grounded in the early identification of dysfunctional romantic beliefs and the restructuring of cognitive schemas related to love and emotional bonding.

The objectives of this study were (1) to identify the manifestations of psychological violence and attitudes toward love in young university students within intimate partner relationships; (2) to examine the relationship between psychological violence and love attitudes; and (3) to determine the statistical contribution of love attitudes to explaining the variability in psychological violence.

### Hypotheses

**Hypothesis** **1.** *The different manifestations of psychological violence (devaluation, hostility, indifference, intimidation, imposition of behaviours, blaming, and apparent kindness) and love attitudes (Eros, Ludus, Storge, Pragma, Mania, and Agape) will be present in the sample of university students, showing variability in their levels of expression.*

**Hypothesis** **2.** *Love attitudes will be significantly associated with the different manifestations of psychological violence.*

**Hypothesis** **3.** *Love attitudes will significantly contribute to explaining the variance in psychological violence.*

## 2. Materials and Methods

### 2.1. Participants

The sample consisted of 1118 students from the University of Extremadura (UEx), Spain, of whom 478 were men (42.8%) and 640 were women (57.2%), aged between 17 and 23 years (*M* = 20.17; *SD* = 1.66).

Given the characteristics of the study, the following inclusion criteria were established: (a) having had at least one romantic relationship, either currently or in the past; (b) being between 17 and 23 years of age; (c) having Spanish as a native language or demonstrating a proficiency level equivalent to bilingual competence; and (d) providing voluntary informed consent to participate in the study. On the other hand, the exclusion criteria were defined as (a) the presence of incomplete data in any of the administered scales; (b) the detection of inconsistent or random response patterns that could compromise data validity; and (c) never having had a romantic relationship.

In this regard, it should be noted that the initial sample consisted of 1255 participants. However, 137 were excluded from the final analysis for not meeting the established criteria: two due to incomplete questionnaires and the remainder for never having had a romantic relationship. Consequently, the final sample comprised 1118 participants.

Participants were enrolled in various undergraduate programmes at the Badajoz campus of the UEx, including Psychology, Medicine and Health Sciences, Business Administration and Management, Economics and Labour Relations and Human Resources, Sciences, Documentation and Communication Sciences, and Engineering.

A probabilistic cluster sampling method was employed, using class groups from the different degree programmes as the sampling units. Once the complete set of available clusters had been identified, their selection was carried out through a random procedure based on the full list of groups. In this way, the class groups that participated in the study were selected, taking advantage of the natural groupings existing within the university population. The final sample consisted of students who voluntarily agreed to complete the questionnaires, which contributed to ensuring the quality and reliability of the data collected.

No economic or academic incentives were offered for participation, and both voluntariness and informed consent were ensured for all participants.

### 2.2. Instruments

The instruments used to evaluate the research variables were as follows:

- *Questionnaire on Psychological Violence in Intimate Partner Relationships (CVPPar)* (Pérez-Díaz et al., 2025): The instrument employed allows for the assessment of psychological violence in intimate partner relationships based on the indicators proposed by Taverniers (2001). It consists of 46 Likert-type items designed to identify manifestations of psychological violence across seven factors: (1) Devaluation, with 10 items (“*My partner rejects my displays of affection*”); (2) Hostility, with six items (“*My partner recalls things from the past in order to hurt me*”); (3) Indifference, with four items (“*My partner doesn’t take my feelings into account*”); (4) Intimidation, with six items (“*My partner usually comes too close to me when reproaching me for something*”); (5) Imposition of behaviour, with 12 items (“*My partner believes that she/he has the right to force me to do things*”); (6) Blaming, with six items (“*My partner accuses me of constantly imagining things that do not happen*”); and (7) Apparent Kindness, with two items (“*My partner says he/she doesn’t like me going places without him/her because he/she is trying to protect me*”). The scale includes five response options reflecting the degree of agreement or disagreement with each statement: 0 (never), 1 (rarely), 2 (sometimes), 3 (frequently), and 4 (always). Scoring is obtained by summing responses within each factor to generate partial scores for the different manifestations of psychological violence; higher scores indicate a greater presence of psychological violence. In order to assess the construct validity of the instrument in the present study sample, a Confirmatory Factor Analysis (CFA) was conducted, the results of which supported the seven-dimensional factorial structure. The model showed an adequate fit to the data (CFI = 0.96; TLI = 0.95; RMSEA = 0.04), confirming its validity in the analysed sample. Internal consistency (Cronbach’s alpha) was 0.96 for the overall scale, 0.84 for Devaluation, 0.88 for Hostility, 0.81 for Indifference, 0.80 for Intimidation, 0.86 for Imposition of behaviour, 0.79 for Blaming, and 0.62 for Apparent Kindness.
- *Short Love Attitudes Scale (LAS)* (S. S. Hendrick et al., 1998), adapted into Spanish by Ubillos and Barrientos (2001): The instrument allows for the exploration of participants’ attitudes toward love, aiming to develop an individual profile based on the scores obtained across the different love styles proposed by Lee (1973). It consists of 18 Likert-type items distributed across six dimensions: (1) Eros, with three items (“*I feel that my partner and I are made for each other*”); (2) Ludus, with three items (“*At times, I have had to conceal things about my previous partners*”); (3) Storge, with three items (“*The deepest kind of love emerges from a long-standing friendship*”); (4) Pragma, with three items (“*An important criterion when choosing a partner is whether he/she would be a good parent*”); (5) Mania, with three items (“*When I am in love, I struggle to concentrate on anything other than my partner*”); and (6) Agape, with three items (“*I would rather suffer myself than see my partner suffer*”). The scale includes five response options reflecting the degree of agreement or disagreement with each statement: 1 (strongly disagree), 2 (disagree), 3 (neither agree nor disagree), 4 (agree), and 5 (strongly agree). Scoring is obtained by summing the responses within each factor to generate partial scores for each love style. Higher scores indicate a greater degree of agreement with or identification with the corresponding love style. In addition, a CFA was conducted, the results of which supported the six-factor structure. The model showed satisfactory fit indices (CFI = 0.95; TLI = 0.93; RMSEA = 0.06), confirming the adequacy of the factorial model in the present study sample. Internal consistency (Cronbach’s alpha) was 0.83 for the overall scale, 0.87 for Eros, 0.80 for Ludus, 0.79 for Storge, 0.82 for Mania, 0.68 for Pragma, and 0.88 for Agape.

### 2.3. Procedure

In the initial phase, the Faculties of the Badajoz Campus of the University of Extremadura were selected, and the necessary authorizations were obtained from the Academic Secretariats.

Students were provided with prior information regarding the study’s objectives, participation conditions, and their right to withdraw at any time. All participants gave informed consent prior to the administration of the instruments. Data collection was carried out through the in-person administration of the instruments in paper-and-pencil format during group sessions held in both morning and afternoon schedules. The questionnaires were administered by previously trained members of the research team, without the direct involvement of the teaching staff responsible for the courses, in order to minimise potential influences arising from the presence of authority figures.

Anonymity and confidentiality were ensured through the assignment of an individual numerical code not linked to personal identity. Participants were also explicitly informed that their responses would be treated confidentially and used exclusively for research purposes, with no academic consequences. These conditions were established to reduce potential social desirability bias and to encourage honest responding. The total duration of the assessment ranged between 35 and 40 min.

All procedures were carried out in accordance with the ethical standards of the University of Extremadura (Ref: 92/2020), as well as with the principles of the 1964 Declaration of Helsinki and its subsequent amendments, or comparable ethical standards.

### 2.4. Data Analysis

Data analysis was performed using the Statistical Package for the Social Sciences (SPSS), version 27.0.

In the first stage, a descriptive analysis was conducted on psychological violence manifestations and love attitudes in romantic relationships among university students, in order to examine the overall distribution of the study variables. Subsequently, these statistical assumptions were assessed: normality of the data using the Kolmogorov–Smirnov test (*p* > 0.05), randomness of the sample using the Runs test (*p* > 0.05), and homoscedasticity using Levene’s test (*p* > 0.05). The confirmation of these assumptions allowed for the application of parametric tests.

A correlational analysis (Pearson’s r) was performed to examine the relationship between psychological violence manifestations and love attitudes, with the aim of identifying potential associations. This was followed by a multiple linear regression analysis to determine the contribution of love attitudes in explaining the variance of the different manifestations of psychological violence. In all regression analyses, standardised coefficients (β) allowed for the direct comparison of the relative magnitude of the associations between the variables included in each model.

## 3. Results

### 3.1. Descriptive Analysis

Regarding the descriptive analysis, the CVPPar results indicate that, in the total sample, manifestations of psychological violence varied in prevalence and intensity. Within the CVPPar, indifference emerged as the dimension with the highest mean score. In the LAS, the love styles most strongly endorsed by participants were Eros, Mania, and Ludus.

The results show the scores in Table 1.

### 3.2. Inferential Analysis

Table 2 presents the results of the correlational analysis examining the relationship between psychological violence manifestations and different love styles in the romantic relationships of university students.

The results indicate significant correlations between psychological violence manifestations and love attitudes in romantic relationships. In terms of effect size, correlations ranged predominantly from small to moderate magnitudes (*r* ≈ 0.07–0.42), showing a generally consistent pattern.

The Eros love style was significantly associated with several manifestations of psychological violence, showing correlations of small to moderate magnitude, with notable values for devaluation (*r* = 0.379, *p* < 0.01), hostility (*r* = 0.257, *p* < 0.01), intimidation (*r* = 0.346, *p* < 0.01), imposition of behaviour (*r* = 0.319, *p* < 0.01), blaming (*r* = 0.336, *p* < 0.01), and apparent kindness (*r* = 0.219, *p* < 0.01).

The Storge style showed low-magnitude associations in the sample, with significant relationships observed for devaluation (*r* = 0.143, *p* < 0.05) and intimidation (*r* = 0.171, *p* < 0.05), whereas the remaining associations did not reach statistical significance.

In the case of Ludus, correlations were systematically higher than those observed for Storge and Pragma, ranging from small to moderate magnitudes. Notable associations were found for devaluation (*r* = 0.362, *p* < 0.01), hostility (*r* = 0.417, *p* < 0.01), indifference (*r* = 0.323, *p* < 0.01), intimidation (*r* = 0.293, *p* < 0.01), imposition of behaviour (*r* = 0.279, *p* < 0.01), blaming (*r* = 0.369, *p* < 0.01), and apparent kindness (*r* = 0.290, *p* < 0.01).

Pragma showed positive low-magnitude associations with devaluation (*r* = 0.183, *p* < 0.05), hostility (*r* = 0.186, *p* < 0.05), indifference (*r* = 0.178, *p* < 0.05), imposition of behaviours (*r* = 0.268, *p* < 0.01), and apparent kindness (*r* = 0.183, *p* < 0.05), whereas no significant associations were observed with intimidation or blaming.

Consistently, Mania showed positive correlations of moderate magnitude with most manifestations of psychological violence, with notable associations for devaluation (*r* = 0.374, *p* < 0.01), hostility (*r* = 0.342, *p* < 0.01), indifference (*r* = 0.308, *p* < 0.01), intimidation (*r* = 0.395, *p* < 0.01), imposition of behaviours (*r* = 0.259, *p* < 0.01), blaming (*r* = 0.408, *p* < 0.01), and apparent kindness (*r* = 0.310, *p* < 0.01).

Finally, Agape showed low-magnitude associations with devaluation (*r* = 0.261, *p* < 0.01), hostility (*r* = 0.196, *p* < 0.05), intimidation (*r* = 0.223, *p* < 0.01), and blaming (*r* = 0.190, *p* < 0.05), while no significant associations were observed with indifference, imposition of behaviours, or apparent kindness.

Overall, the results reveal a consistent pattern of positive correlations between love attitudes, particularly Eros, Ludus, and Mania, and most manifestations of psychological violence.

Table 3 presents the results of the multiple linear regression analysis conducted to determine the statistical contribution of love attitudes in explaining the variability of psychological violence in university students’ romantic relationships.

The regression analysis results indicated that love attitudes are significantly associated with manifestations of psychological violence. Overall, the models showed explained variance values ranging from 34% to 43%, suggesting a moderate and stable explanatory capacity.

For devaluation, the model explained 35.4% of the variance (R^2^ = 0.354), with significant moderate associations observed for Mania (β = 0.312; *p* < 0.01), Eros (β = 0.281; *p* < 0.01), and Ludus (β = 0.242; *p* < 0.01), along with smaller effects for Storge (β = 0.184; *p* < 0.01) and Pragma (β = 0.177; *p* < 0.01), whereas Agape showed a small contribution (β = 0.101; *p* < 0.05). For hostility, the model explained 36.2% of the variance (R^2^ = 0.362), with significant associations emerging for Mania (β = 0.331; *p* < 0.01), Eros (β = 0.271; *p* < 0.01), and Ludus (β = 0.212; *p* < 0.01), while Agape showed a smaller effect size (β = 0.112; *p* < 0.01).

Regarding indifference, the model explained 34.7% of the variance (R^2^ = 0.347), with significant moderate associations observed for Mania (β = 0.304; *p* < 0.01) and Ludus (β = 0.279; *p* < 0.01), along with a relevant contribution from Pragma (β = 0.226; *p* < 0.01), whereas Agape showed a small contribution (β = 0.086; *p* < 0.05). The remaining predictors did not reach statistical significance. With respect to intimidation, the model explained 43.2% of the variance (R^2^ = 0.432), with significant moderate associations identified for Mania (β = 0.351; *p* < 0.01), Eros (β = 0.291; *p* < 0.01), and Ludus (β = 0.270; *p* < 0.01), along with additional contributions from Agape (β = 0.165; *p* < 0.01) and Storge (β = 0.143; *p* < 0.01).

For imposition of behaviours, the model explained 41.1% of the variance (R^2^ = 0.411), showing significant moderate associations with Eros (β = 0.302; *p* < 0.01), Mania (β = 0.287; *p* < 0.01), and Ludus (β = 0.256; *p* < 0.01), whereas Pragma showed a smaller contribution (β = 0.153; *p* < 0.01), and the remaining predictors displayed weaker effects, namely Storge (β = 0.094; *p* < 0.05) and Agape (β = 0.093; *p* < 0.05). For blaming, the model explained 39.3% of the variance (R^2^ = 0.393), with significant moderate associations observed for Mania (β = 0.348; *p* < 0.01), Ludus (β = 0.287; *p* < 0.01), and Eros (β = 0.263; *p* < 0.01), along with additional effects of Agape (β = 0.175; *p* < 0.01) and Pragma (β = 0.093; *p* < 0.05).

Finally, regarding apparent kindness, the model explained 40.8% of the variance (R^2^ = 0.408), with significant moderate associations observed for Mania (β = 0.291; *p* < 0.01) and Eros (β = 0.274; *p* < 0.01), as well as contributions from Pragma (β = 0.215; *p* < 0.01) and Ludus (β = 0.207; *p* < 0.01), whereas Agape showed a small but significant effect (β = 0.084; *p* < 0.05).

The results reveal a robust pattern in which the Mania, Eros, and Ludus love styles show the strongest associations with the different manifestations of psychological violence, maintaining a consistent and stable effect structure.

## 4. Discussion

This study contributes to a deeper understanding of the manifestations of psychological violence in intimate partner relationships and their association with love attitudes among university students. Consistent with previous research, the findings confirm that psychological violence manifests in multiple ways and encompasses a wide range of behaviours, reinforcing its multidimensional nature and operational complexity (Martín & Moral, 2019; Villavicencio-Aguilar & Jaramillo-Paladinez, 2020). Among these expressions, indifference stands out as one of the most prevalent behaviours, supporting evidence that identifies it as a particularly harmful form of violence. Its subtle nature facilitates its invisibility, contributing to perceived emotional neglect, diminished self-esteem, and the consolidation of maladaptive cognitive schemas (Dodaj et al., 2020; Echeburúa & Muñoz, 2017; Fernández-Fuertes et al., 2020; Salvazán et al., 2014; Tester, 2002).

Regarding attitudes toward love, the findings reveal the coexistence of different love styles within the sample. Overall, there is a notable presence of the Eros, Ludus, and Mania styles, alongside the concurrence of styles characterised by a stronger orientation toward relational stability, such as Storge, Pragma, and Agape. These results are consistent with the literature describing early romantic experiences as idealised contexts in which passion and physical and emotional intimacy are prioritised over components such as commitment or long-term stability (Clemente et al., 2020; Liu et al., 2024; Neto, 2001). In this regard, several authors argue that youth represents a stage of emotional transition characterised by greater affective and sexual freedom, as well as the absence of consolidated adult responsibilities. This context fosters the development of unstable and fluctuating relationships, alternating between committed partnerships and casual encounters, in line with the pursuit of experiences that meet the expectations typical of this developmental stage (Cohen et al., 2003; Shams & Atta, 2024; Yancey & Eastman, 1995).

Thus, these findings are consistent with the notion that love attitudes do not constitute isolated constructs, but rather sociocognitive schemas shaped through interaction with relational and sociocultural contexts, which may influence how certain dynamics within the couple are interpreted and maintained. Within this framework, the results support the relevance of these variables for understanding the variability of psychological violence in university populations (Fermani et al., 2019; Lantagne & Furman, 2017).

Regarding the second and third hypotheses, the results show significant relationships between love attitudes and the different manifestations of psychological violence, confirming their role as relevant variables in explaining this phenomenon. Consistently, the Eros, Ludus, and Mania styles exhibit the most robust associations, whereas Storge, Pragma, and Agape show more specific and lower-intensity relationships.

The Eros style was associated with a broad range of manifestations of psychological violence, including devaluation, hostility, intimidation, imposition of behaviours, blaming, and apparent kindness. This pattern is consistent with the literature suggesting that idealised representations of romantic love may generate frustration when reality does not align with these expectations, giving rise to manifestations of psychological violence that often go unnoticed due to social normalisation (Flores et al., 2019; Hernández et al., 2020; Jiménez-Picón et al., 2023). In this regard, the ideology of romantic love has been identified as a cultural framework that may contribute to the implicit legitimization of controlling behaviours or relational imbalances in young people’s intimate relationships (Bonilla-Algovia et al., 2021; Fedele et al., 2019; Márquez et al., 2020).

Similarly, the Mania style showed significant correlations and a stronger association with most forms of psychological violence. These findings are consistent with previous research linking this style to patterns of emotional dependence, anxious attachment, affective instability, and impulsivity, variables identified as relevant in dysfunctional relational dynamics (Alcalá et al., 2021; Blanchard & Fino, 2023; Robles et al., 2021). Other studies, including those by Oldac et al. (2025) and Yıldız and Eldeleklioğlu (2021), indicate that the Mania style is closely associated with domination and control, showing a consistent relationship with violent behaviours. Moreover, this affective pattern may function as a mechanism for maintaining the relationship through strategies of isolation and emotional dependence (Honari & Saremi, 2015; Kumul, 2019).

The Ludus style also shows significant associations with multiple forms of psychological violence, including devaluation, hostility, indifference, intimidation, imposition of behaviours, blaming, and apparent kindness, as well as significant relationships with several of these dimensions. These findings are consistent with the literature linking this style to relational dynamics characterised by lower emotional involvement, greater instrumentalization of the relationship, and potential manipulative strategies (Altınok & Kılıç, 2020; Rafiezadeh & Zarehneyestanak, 2021; Vu, 2020).

Regarding Storge, the results show more specific associations, particularly its relationship with devaluation and intimidation. These findings suggest that perceptions of relational stability and trust may coexist with dynamics in which controlling behaviours or progressive restrictions of autonomy are normalised, especially in contexts of high emotional involvement (Díaz et al., 2019; Jiménez-Picón et al., 2023; Messing et al., 2021).

Regarding Pragma, significant associations were observed with several manifestations of psychological violence, including devaluation, hostility, indifference, imposition of behaviours, and apparent kindness. These results suggest that a pragmatic orientation toward relationships, focused on compatibility and stability, may be associated with relational dynamics in which certain forms of control or interpersonal imbalance are tolerated or justified.

The Agape style also shows significant correlations with several manifestations of psychological violence, such as devaluation, hostility, intimidation, and blaming, as well as associations with devaluation, hostility, indifference, intimidation, imposition of behaviours, blaming, and apparent kindness. This finding suggests that, in certain contexts, an altruistic orientation within the relationship may be associated with relational dynamics in which care and self-sacrifice can contribute to the acceptance or justification of dysfunctional behaviours.

In explanatory terms, the regression models indicate that love attitudes account for a relevant and consistent proportion of the variability in psychological violence, reinforcing their role as meaningful constructs in understanding this phenomenon in a university context. Specifically, the results consistently highlight Mania, Eros, and Ludus as the styles with the greatest relative weight in their association with the different manifestations of psychological violence. Pragma, Storge, and Agape also showed significant effects in explaining variance, although to a lesser extent depending on the specific indicator.

These findings have relevant applied implications, as they suggest the usefulness of incorporating the analysis of love attitudes into the design of preventive interventions. In particular, they highlight the importance of developing early psychoeducational programmes with a gender perspective, aimed at promoting relationships based on equality and emotional well-being. This preventive approach is especially relevant during the stages in which early romantic relationships are being consolidated.

Finally, this study presents certain limitations that should be taken into consideration. The sample, composed exclusively of university students from a single university, restricts the generalisability of the findings to other academic, sociocultural contexts, or age groups. Likewise, the cross-sectional design precludes the establishment of causal relationships and the temporal ordering of variables, limiting the analysis of their temporal evolution.

Another limitation arises from the use of self-report measures based on Likert-type scales. This may lead to response biases, such as social desirability or a tendency toward mid-point responses, as well as common method bias.

Additionally, it is necessary to note the low reliability obtained for the apparent kindness factor. This dimension is composed of a reduced number of items and, from a theoretical standpoint, is essential for capturing the manipulation of reality proposed by Taverniers (2001). Simulating love and interest in order to justify violent behaviours constitutes an apparent act of kindness that conceals the manipulation of reality (Martínez et al., 2024). In this regard, despite its limitations, its inclusion is considered relevant due to its theoretical and clinical importance.

Nevertheless, these aspects open new avenues for future research. It is recommended to conduct longitudinal studies that allow for the evaluation of the stability and changes in the variables over time, also incorporating sociodemographic factors such as social environment, relationship duration, or prior relational experience, in order to provide a broader understanding of the interaction between attitudes toward love and psychological violence.

## 5. Conclusions

This study highlights the importance of examining psychological violence within the context of intimate partner relationships and its association with love attitudes among university populations. The findings show that the different manifestations of psychological violence and love styles coexist in the sample and are systematically related, displaying consistent patterns of association. Likewise, love attitudes make a significant contribution to explaining variability in psychological violence, although this does not imply the existence of causal relationships.

Overall, these results reinforce previous evidence and expand the empirical understanding of the phenomenon within the university population, particularly in relation to styles such as Eros, Ludus, and Mania.

Likewise, from a theoretical perspective, these findings contribute to a clearer delineation of the sociocognitive processes involved in couple dynamics by considering love attitudes as schemas that guide the interpretation and regulation of affective interactions. In this sense, their inclusion in the study of psychological violence helps advance toward more integrative explanatory models that better capture the complexity of the phenomenon.

Thus, the evidence obtained supports the relevance of developing more specific future research lines, including longitudinal designs that allow for the examination of the temporal evolution of these variables, dyadic designs incorporating both members of the couple, and multi-informant approaches aimed at reducing potential biases derived from self-report measures.

Finally, future research in this area is encouraged to further explore the psychosocial mechanisms linking love styles to the different manifestations of psychological violence in young couples, in order to improve the understanding of these relational processes and strengthen the empirical basis for the design of effective preventive interventions.

## Figures and Tables

**Table 1:** Descriptive statistics.

	*M*	*SD*
Devaluation	23.47	3.20
Hostility	10.51	2.83
Indifference	10.13	2.06
Intimidation	10.64	2.54
Imposition of behaviour	17.54	5.18
Blaming	11.61	2.87
Apparent kindness	2.73	1.67
Eros	11.75	2.76
Storge	9.36	2.23
Ludus	10.33	2.27
Pragma	9.23	2.22
Mania	11.43	2.83
Agape	9.36	2.11

**Table 2:** Correlational analysis between manifestations of psychological violence and attitudes toward love.

	Eros	Storge	Ludus	Pragma	Mania	Agape
Devaluation	0.379 **	0.143 *	0.362 **	0.183 *	0.374 **	0.261 **
Hostility	0.257 **	0.049	0.417 **	0.186 *	0.342 **	0.196 *
Indifference	0.077	0.068	0.323 **	0.178 *	0.308 **	0.089
Intimidation	0.346 **	0.171 *	0.293 **	0.065	0.395 **	0.223 **
Imposition of behaviour	0.319 **	0.042	0.279 **	0.268 **	0.259 **	0.117
Blaming	0.336 **	0.058	0.369 **	0.056	0.408 **	0.190 *
Apparent kindness	0.219 **	0.021	0.290 **	0.183 *	0.310 **	0.072

Note: Pearson’s correlation coefficient * *p* < 0.05; ** *p* < 0.01.

**Table 3:** Multiple linear regression analysis between love attitudes and manifestations of psychological violence.

	Dvl		Hst		Ind		Int		Iob		Blm		Apk	
	β	t	β	t	β	t	β	t	β	t	β	t	β	t
Eros	0.281	7.403 **	0.271	6.521 **	0.055	1.452	0.291	7.605 **	0.302	7.811 **	0.263	7.196 **	0.274	7.246 **
Storge	0.184	4.201 **	0.075	1.943	0.063	1.651	0.143	3.632 **	0.094	2,205 *	0.072	1.803	0.072	1.921
Ludus	0.242	6.821 **	0.212	5.906 **	0.279	7.203 **	0.270	7.115 **	0.256	6.635 **	0.287	7.403 **	0.207	5.523 **
Pragma	0.177	4.311 **	0.056	1.457	0.226	5.672 **	0.053	1.332	0.153	3.797 **	0.093	2.405 *	0.215	5.405 **
Mania	0.312	8.651 **	0.331	9.101 **	0.304	8.214 **	0.351	9.473 **	0.287	7.909 **	0.348	9.051 **	0.291	8.101 **
Agape	0.101	2.721 *	0.112	3.101 **	0.086	2.244 *	0.165	4.260 **	0.093	2.452 *	0.175	4.622 **	0.084	2.113 *
R^2^	0.354		0.362		0.347		0.432		0.411		0.393		0.408	

Note: β = standardised coefficients; t = t statistic; Dvl, Devaluation; Hst, Hostility; Ind, Indifference; Int, Intimidation; Iob, Imposition of behaviour; Blm, Blaming; Apk, Apparent kindness; * *p* < 0.05; ** *p* < 0.01.

## Data Availability

The data presented in this study are available on request from the corresponding author. The data are not publicly available due to privacy reasons and ethical restrictions.
